# Socioeconomic inequalities in childhood-to-adulthood BMI tracking in three British birth cohorts

**DOI:** 10.1038/s41366-019-0387-z

**Published:** 2019-06-05

**Authors:** Tom Norris, David Bann, Rebecca Hardy, William Johnson

**Affiliations:** 1 School of Sport, Exercise and Health Sciences, Loughborough University, Loughborough, LE11 3TU UK; 2 Centre for Longitudinal Studies, UCL Institute of Education, University College London, 20 Bedford Way, London, WC1H 0AL UK; 3 MRC Unit for Lifelong Health and Ageing at UCL, 33 Bedford Place, London, WC1B 5JU UK; 4 School of Sport, Exercise and Health Sciences, Loughborough University, Loughborough, LE11 3TU UK

**Keywords:** Risk factors, Epidemiology

## Abstract

**Background:**

Body mass index (BMI) tracks from childhood-to-adulthood, but the extent to which this relationship varies across the distribution and according to socio-economic position (SEP) is unknown. We aimed to address this using data from three British cohort studies.

**Methods:**

We used data from: 1946 National Survey of Health and Development (NSHD, *n* = 2470); 1958 National Child Development Study (NCDS, *n* = 7747); 1970 British Cohort Study (BCS, *n* = 5323). BMI tracking between 11 and 42 years was estimated using quantile regression, with estimates reflecting correlation coefficients. SEP disparities in tracking were investigated using a derived SEP variable based on parental education reported in childhood. This SEP variable was then interacted with the 11-year BMI z-score.

**Results:**

In each cohort and sex, tracking was stronger at the upper end of the distribution of BMI at 42 years. For example, for men in the 1946 NSHD, the tracking estimate at the 10th quantile was 0.31 (0.20, 0.41), increasing to 0.71 (0.61, 0.82) at the 90th quantile. We observed no strong evidence of SEP inequalities in tracking in men in the 1946 and 1958 cohorts. In the 1970 cohort, however, we observed tentative evidence of stronger tracking in low SEP groups, particularly in women and at the higher end of the BMI distribution. For example, women in the 1970 cohort from low SEP backgrounds had tracking coefficients at the 50th, 70th, and 90th quantiles, which were 0.05 (−0.04; 0.15), 0.19 (0.06; 0.31), and 0.22 (0.02; 0.43) units higher, respectively, than children from high SEP groups.

**Conclusion:**

Tracking was consistently stronger at the higher quantiles of the BMI distribution. We observed suggestive evidence for a pattern of greater BMI tracking in lower (compared to higher) SEP groups in the more recently born cohort, particularly in women and at the higher end of the BMI distribution.

## Introduction

The obesity epidemic is a serious global public health concern. In 2016 the worldwide prevalence of adult overweight or obesity (according to body mass index (BMI)) was 39% [1], with higher prevalence rates observed in high-income countries [2]. The 2016 Health Survey for England (HSE) for example, reported that 26% and 27% of adult men and women, respectively, were obese, with a further 40% of men and 30% of women being classified as overweight. Children are not exempt from this epidemic and data from the 2017–2018 National Child Measurement Programme (NCMP) revealed that 9.5% of children in England were obese when entering primary school (ages 4–5 years), increasing to 20.1% when entering secondary school (ages 10–11 years) [3]. An analysis using data from five UK birth cohorts [4] also revealed a trend towards an earlier onset of obesity in more recent cohorts and thus a greater lifetime exposure. For example, more recently born cohorts had probabilities of childhood overweight/obesity, which were two to three times greater than those for earlier born cohorts.

The assumption underlying the idea that an earlier onset of obesity represents a greater lifetime exposure is based upon the notion that weight tracks over the life course. Studies investigating the tracking of weight have typically identified it using continuous BMI and/or BMI categories (e.g. normal weight, overweight, and obesity) [5–8]. Thus, the risk of a heavy child being an adult who is overweight or obese (i.e. tracking) is greater amongst children who are overweight/obese [5, 9]. While it is likely that children at the extremes of the distribution remain at the extremes at a subsequent time point, the evidence of tracking in those not in the extremes (i.e. normal weight or moderately overweight children) is equivocal [5, 10, 11]. When looking at tracking defined using BMI on a continuous scale (as opposed to BMI categories), inconsistent estimates are reported, as summarised in a meta-regression of the reported tracking (correlation) coefficients [12]. However, the use of correlation or linear regression to assess tracking results in an estimate of the tracking only at the mean of the two BMI variables and thus it is provides no understanding of the variability of tracking across the BMI distribution. In the previously mentioned meta-regression, a small positive association was observed between tracking and cohort BMI, with cohorts with higher BMI at baseline showing higher tracking coefficients. However, the evidence supporting the association was weak [12]. Further investigation into whether there is differential tracking depending on an individual’s position within the BMI distribution is therefore warranted. As it has been demonstrated that the obesity epidemic reflects an increasing number of individuals at the upper end of the BMI distribution (i.e. a positive skewing of the BMI distribution) [4, 13–16], it is important to comprehend the likelihood of life course tracking in this increasing population of people.

Socioeconomic disparities in obesity are well established in high-income countries, with systematic reviews highlighting the association between lower socioeconomic position (SEP) and higher BMI and an increased obesity risk, in both childhood and adulthood [17, 18]. In addition to these cross-sectional associations, consistent associations have been observed between childhood SEP and adult BMI [6, 7, 19–21], independent of adult SEP, suggesting that SEP inequalities in BMI and obesity risk may track across the life course. For example, a study using three nationally representative birth cohorts observed large inequalities in adult BMI according to childhood SEP, with increasing inequality in the most recently born cohort [21]. A more recent analysis extended this work by examining SEP inequalities across the range of the BMI distribution (not just the mean) in childhood and adolescence and observed increasing SEP inequalities in BMI at the higher end of the BMI distribution [22]. A small number of studies have observed SEP inequalities in the tracking of BMI, however these have spanned only a short period of the life course [23–25]. We are not aware of any studies which have investigated whether SEP inequalities exist in the tracking of BMI from childhood-to-adulthood.

In summary, there is a need to understand whether there is differential tracking depending on an individual’s position not only within the BMI distribution, but also their socioeconomic position. If, for example tracking is observed to be stronger in lower SEP groups, this would suggest that children who are obese and from disadvantaged backgrounds would be more likely to be obese in adulthood than children who are equally obese but from advantaged backgrounds. In an exploratory analysis using data from three British birth cohort studies, we aimed to examine how childhood-to-adulthood BMI tracking varies across the distribution and according to socio-economic position. In light of recent evidence suggesting increasing SEP inequalities at the upper end of the BMI distribution, we hypothesised that differences between socioeconomic groups would be larger at the higher end of the BMI distribution.

## Methods

### Samples

We used data from three British birth cohort studies. These cohorts have been previously described in detail elsewhere [26–28] and were designed to be nationally representative when initiated in 1946 (MRC National Survey of Health and Development [NSHD]; *n* = 5362 (weighting can be applied in analyses in order to adjust for the sampling procedure), 1958 (National Child Development Study [NCDS]; *n* = 17,416) and 1970 (British Cohort Study [BCS]; *n* = 16,571). All of the studies have received ethical approval and obtained informed parental and/or participant consent; this information is available from the study websites and/or cohort profiles [26–29]

A total of 15,540 participants had complete exposure and outcome data of whom 7874 (52.2%) were male. This sample represents 2,470 participants from the 1946 cohort (76% of those still participating in the cohort at 43 years); 7747 participants from the 1958 cohort (68% of those still participating in the cohort at 42 years); and 5323 participants from the 1970 cohort (representing 54% of those still participating in the cohort at 42 years).

### Body mass index

As described elsewhere [4], serial BMI (kg/m^2^) was derived and harmonised in each study from measured or self-reported weight and height. For this study, we used BMI collected at 11 and 43 years of age in the 1946 cohort, 11 and 42 years in the 1958 cohort and 10 and 42 years in the 1970 cohort. BMI at 42 years in 1958 and 1970 cohorts were based on self-reported height and weight. Hereafter BMI will be referred to as BMI at 11 and BMI at 42.

### Childhood socioeconomic position

Childhood SEP was derived from maternal and paternal education data, namely whether each parent left full-time education at the mandatory leaving age (14 years from 1918, 15 years from 1944, and 16 years from 1972). These dichotomous (0/1) variables were ascertained at birth in the 1958 and 1970 cohorts, and at age 6 years in the 1946 cohort. As a sensitivity analyses, we repeated the analysis using father’s (occupational) social class, reported when the child was 10–11 years, as the indicator of socioeconomic position. The Registrar General’s Social Classes schema was used classify social class and resulted in six social class groups: I (professional), II (managerial and technical), IIIN (skilled nonmanual), IIIM (skilled manual), IV (partly skilled), V (unskilled). The 1990 classification was used for childhood SEP in the 1958 and 1970 cohorts, whereas the 1970 version was used for childhood SEP in the 1946 cohort. Those in the armed forces and not employed were not assigned a social class.

### Statistical analysis

Due to the physiological differences observed between men and women over the life course, an a priori decision was taken to perform all analyses stratified by sex. Furthermore, as it has been observed that the relationship with SEP and BMI may have changed over time [21], we decided to perform analyses in each cohort separately. Standardised BMI values (internal z-scores) were generated ((BMI_individual_ − BMI_mean_)/BMI_SD_) at ages 11 and 42. A categorical variable representing childhood socioeconomic position was derived by combining maternal and paternal education data. This variable comprised three groups: 1 = ‘high educational background’ reflecting parent sets who had both stayed on in full-time education after the mandatory leaving age; 2 = ‘low educational background’ reflecting parent sets who had both left full-time education at the mandatory leaving age; and finally 0 = ‘middle educational background’, representing parent sets in which one of the two stayed on after the mandatory leaving age. For the sensitivity analysis, a similar categorical variable based on childhood social class was derived, again comprising three groups: 1 = ‘high social class’ reflecting those whose fathers were classified as I or II (‘professional’ and ‘managerial and technical’) on the Registrar General’s classification; 2 = ‘low social class’ reflecting those whose fathers were classified as IV or V (‘partly skilled’ and ‘unskilled’, respectively); and finally 0 = ‘middle social class’, representing those whose fathers were classified as IIIN or IIIM (‘skilled manual’ and ‘skilled non-manual’, respectively).

BMI tracking between 11 and 42 years was estimated using a quantile regression of BMI z-score at 42 years on BMI z-scores at 11 years. To investigate tracking across the BMI distribution, we extracted estimates at the 10th, 30th, 50th, 70th and 90th quantiles of the 42-year BMI z-score. As both the 11 and 42-year BMI values had been standardised, the coefficients from these quantile regressions can be interpreted as the correlation coefficients (‘tracking coefficients’) between the two measures, at different quantiles of the 42-year BMI z-score. To determine whether tracking differed across SEP groups, we included the categorical SEP variable, with ‘high educational background’ as the referent group, and its interaction with the 11-year BMI z-score. Models were further adjusted for exact age at the 42-year data collection sweep. We used Stata’s ‘grqreg’ command to plot how tracking changes across the entire outcome distribution, for each SEP group. Shading was added between the lines to provide a visual interpretation of the strength of evidence for the difference between the SEP groups. This was calculated at the midpoint of each quantile (e.g. shading between the 10th and 20th quantiles was based on the strength of evidence at the 15th quantile).

## Results

As shown in Table 1, median BMI in childhood was similar across the three cohorts (1946: 17.0 kg/m^2^ (IQR: 15.8, 18.4), 1958: 17.0 kg/m^2^ (15.8,18.5) and 1970: 16.5 kg/m^2^ (15.5, 17.9)) whereas in adulthood, median BMI increased in the more recently born cohorts (NSHD: 24.8 kg/m^2^ (22.6; 27.5), NCDS: 25.2 kg/m^2^ (23.3; 28.2) and 26.0 kg/m^2^ (23.3; 29.3)). This is reflected in the similar prevalence rate of obesity across the three cohorts at age 11 but an increased prevalence in more recent cohorts at age 42 years.

**Table 1:** Descriptive statistics

Cohort			
	1946 (*n* = 2470)	1958 (*n* = 7747)	1970 (*n* = 5323)
Sex [*n*(%)]			
Male	1249 (50.6)	3860 (49.8)	2594 (48.7)
Female	1221 (49.4)	3887 (50.2)	2729 (51.3)
BMI in childhood (kg/m^2^)	17.0 (15.8; 18.4)	17.0 (15.8; 18.5)	16.5 (15.5; 17.9)
BMI classification in childhood^a^ [*n*(%)]			
Thinness	276 (11.2)	1090 (14.1)	602 (11.3)
Normal weight	1993 (80.7)	5953 (76.8)	4276 (80.3)
Overweight	173 (7.0)	599 (7.7)	426 (8.0)
Obese	28 (1.1)	102 (1.3)	19 (0.4)
Age at childhood BMI measurement (years)	10.8 (10.8; 10.9)	11.3 (11.3; 11.4)	10.2 (10.1; 10.3)
Mother educated beyond mandatory leaving age^b^ [*n*(%)]	699 (28.3)	2078 (26.8)	1960 (36.8)
Father educated beyond mandatory leaving age^b^ [*n*(%)]	765 (31.0)	1826 (23.6)	2046 (38.4)
Number of parents educated beyond mandatory leaving age [*n*(%)]			
2	468 (19.0)	1011 (13.1)	1209 (22.7)
1	528 (21.4)	1882 (24.3)	1558 (29.8)
0	1474 (59.7)	4854 (62.7)	2526 (47.5)
Social class^c^ in childhood [*n*(%)]			
Professional	136 (5.9)	363 (4.9)	280 (5.4)
Intermediate	449 (19.4)	1552 (20.8)	1342 (25.7)
Skilled non-manual	381 (16.5)	807 (10.8)	570 (10.9)
Skilled manual	792 (34.3)	3063 (41.0)	2083 (40.0)
Partly skilled manual	429 (18.6)	1090 (14.6)	648 (12.4)
Unskilled manual	124 (5.4)	599 (8.0)	290 (5.6)
BMI in adulthood (kg/m^2^)	24.8 (22.6; 27.5)	25.2 (22.8; 28.2)	26.0 (23.3; 29.3)
BMI classification in adulthood^d^ [*n*(%)]			
Thinness	33 (1.3)	79 (1.0)	60 (1.1)
Normal weight	1255 (50.8)	3602 (46.5)	2159 (40.6)
Overweight	876 (35.5)	2827 (36.5)	1976 (37.1)
Obese	306 (12.4)	1239 (16.0)	1128 (21.2)
Age at adulthood BMI measurement (years)	43.5 (43.3; 43.6)	41.9 (41.8; 42.1)	42.4 (42.3; 42.6)

^a^According to the IOTF classifications

^b^14 years from 1918, 15 years from 1944, and 16 years from 1972

^c^According to Registrar General Social Class classification

^d^According to WHO criteria; continuous variables summarised using median and interquartile range

### Tracking across the BMI distribution

A pattern of greater tracking at higher quantiles of the BMI distribution was observed consistently across the cohorts and sexes. For example, unadjusted tracking estimates at the 10th quantile for men were 0.31 (95% CI: 0.20; 0.41) in the 1946 cohort, 0.30 (0.26; 0.34) in the 1958 and 0.24 (0.19; 0.30) in the 1970 cohort (supplementary table 1). These estimates increased to 0.71 (0.61; 0.82), 0.62 (0.53; 0.71) and 0.56 (0.47; 0.65), respectively, at the 90th quantile.

### Tracking by socioeconomic position

#### Men

**Fig. 1:**
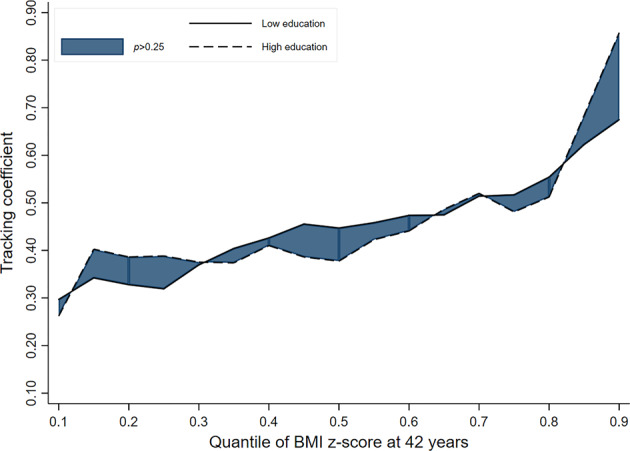
BMI tracking in ‘high educational background’ and ‘low educational background’ groups between ages 11–42 years in men in the 1946 cohort (*n* = 1249)

**Fig. 2:**
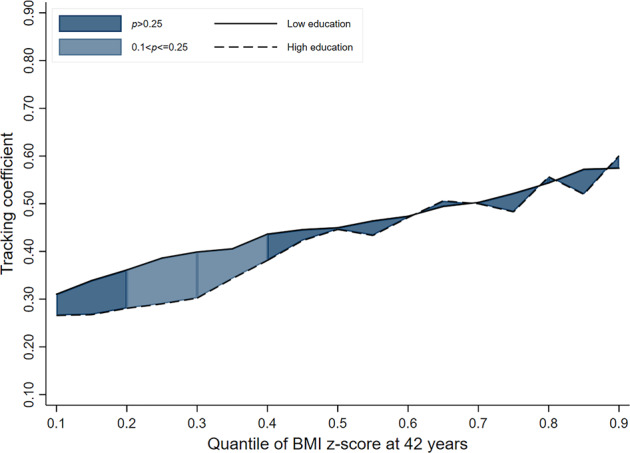
BMI tracking in ‘high educational background’ and ‘low educational background’ groups between ages 11–42 years in men in the 1958 cohort (*n* = 3860)

**Fig. 3:**
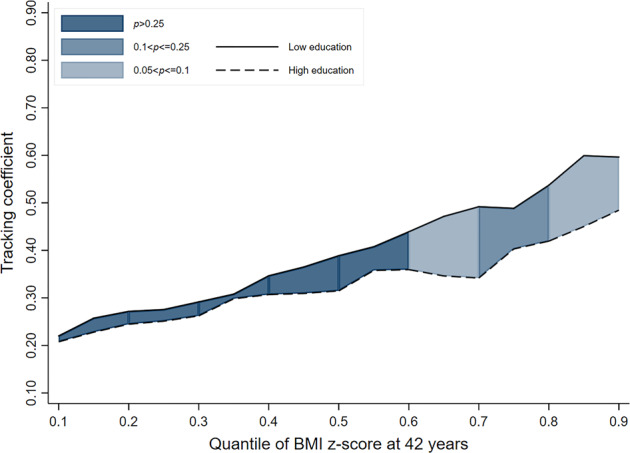
BMI tracking in ‘high educational background’ and ‘low educational background’ groups between ages 11–42 years in men in the 1970 cohort (*n* = 2594)

In the 1946 and 1958 cohorts, there was no evidence to indicate that socioeconomic inequalities exist in child-adult BMI tracking (i.e. overlapping lines for ‘low’ and ‘high’ educational background groups) (Table 2 & Figs. 1–3). While a pattern of stronger tracking in those men from the ‘low educational background’ group (relative to high) may be emerging in the most recent cohort (i.e. line for ‘low educational background’ consistently higher than that for ‘high educational background’), the strength of evidence for this was inconsistent. For example, tracking coefficients in the 1970 cohort at the 50th, 70th and 90th quantiles were 0.09 (−0.01; 0.19, *p*_(difference)_ = 0.085), 0.17 (0.06; 0.29, *p*_(difference)_ = 0.004) and 0.11 (−0.09; 0.27, *p*_(difference)_ = 0.337) higher in those from the ‘low educational background’ group (vs high) (Table 2).

**Table 2:** BMI tracking between ages 11–42 years in men by SEP group (defined using parental education) and cohort, estimated using quantile regression

Cohort									
	1946 NSHD (*n* = 1249)			1958 NCDS (*n* = 3860)			1970 BCS (*n* = 2594)		
	*β*	95% CI	*p*_(diff to ‘High’)_	*β*	95% CI	*p*_(diff to ‘High’)_	*β*	95% CI	*p*_(diff to ‘High’)_
Quantile 0.1									
High education (ref)	0.26	0.12; 0.40	–	0.26	0.15; 0.38	–	0.19	0.08; 0.30	–
Middle education	0.31	0.14; 0.47	0.515	0.27	0.15; 0.39	0.987	0.25	0.16; 0.45	0.344
Low education	0.30	0.22; 0.38	0.635	0.31	0.25; 0.37	0.485	0.22	0.13; 0.31	0.397
Quantile 0.3									
High education (ref)	0.39	0.26; 0.51	–	0.30	0.20; 0.40	–	0.27	0.19; 0.36	–
Middle education	0.52	0.27; 0.76	0.094	0.40	0.32; 0.47	0.112	0.33	0.26; 0.40	0.327
Low education	0.37	0.25; 0.49	0.837	0.40	0.35; 0.45	0.069	0.29	0.22; 0.36	0.887
Quantile 0.5									
High education (ref)	0.39	0.26; 0.52	–	0.45	0.35; 0.54	–	0.30	0.22; 0.39	–
Middle education	0.47	0.29; 0.64	0.388	0.45	0.35; 0.55	0.996	0.39	0.30; 0.48	0.122
Low education	0.45	0.34; 0.55	0.466	0.45	0.41; 0.49	0.997	0.39	0.29; 0.49	0.085
Quantile 0.7									
High education (ref)	0.52	0.39; 0.64	–	0.50	0.39; 0.61	–	0.32	0.21; 0.42	–
Middle education	0.49	0.38; 0.60	0.772	0.55	0.48; 0.62	0.321	0.46	0.35; 0.56	0.01
Low education	0.51	0.43; 0.60	0.963	0.50	0.43; 0.58	0.985	0.49	0.42; 0.57	0.004
Quantile 0.9									
High education (ref)	0.86	0.61; 1.12	–	0.59	0.40; 0.78	–	0.49	0.33; 0.64	–
Middle education	0.61	0.42; 0.80	0.136	0.68	0.53; 0.83	0.425	0.47	0.34; 0.59	0.91
Low education	0.67	0.50; 0.85	0.188	0.57	0.48; 0.67	0.835	0.60	0.46; 0.74	0.337

In analyses classifying childhood SEP based on father’s occupational social class, a similar pattern was observed, with the strongest evidence observed in the most recent cohort in the upper region of the BMI distribution (Supplementary Table 2 & Supplementary Figs. 1–3).

#### Women

**Fig. 4:**
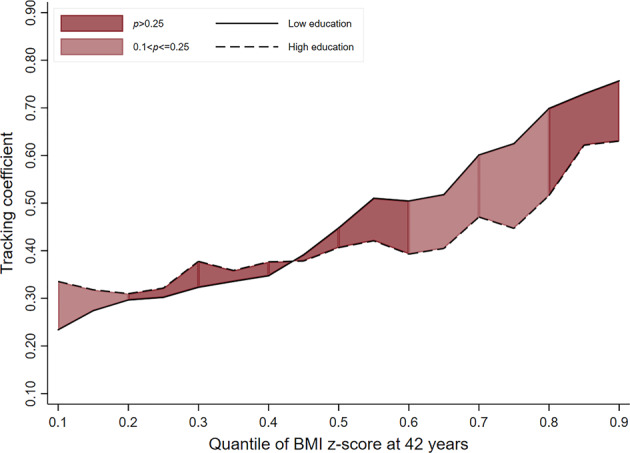
BMI tracking in ‘high educational background’ and ‘low educational background’ groups between ages 11–42 years in women in the 1946 cohort (*n* = 1221)

**Fig. 5:**
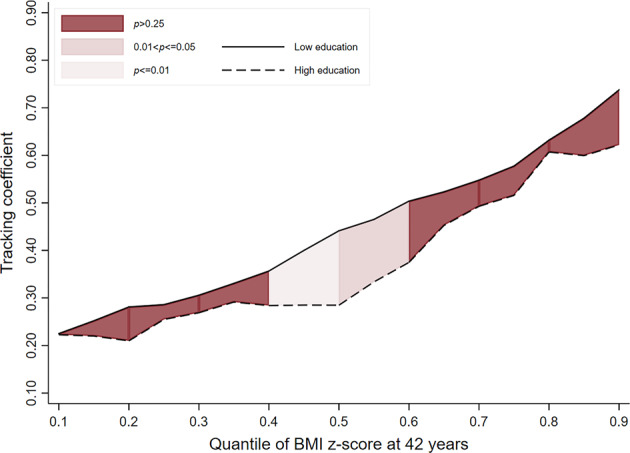
BMI tracking in ‘high educational background’ and ‘low educational background’ groups between ages 11–42 years in women in the 1958 cohort (*n* = 3887)

**Fig. 6:**
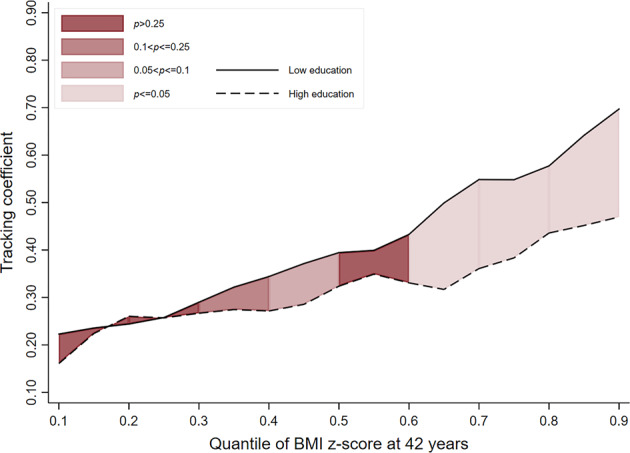
BMI tracking in ‘high educational background’ and ‘low educational background’ groups between ages 11–42 years in women in the 1970 cohort (*n* = 2729)

In women, only in the most recent cohort, and at the higher end of the BMI distribution, was moderate to strong evidence observed. For example, women in the 1970 cohort from ‘low educational backgrounds’ had tracking coefficients at the 50th, 70th and 90th quantiles which were 0.06 (−0.04; 0.15, *p*_(difference)_ = 0.269), 0.19 (0.06; 0.31, *p*_(difference)_ = 0.003) and 0.22 (0.02; 0.43, *p*_(difference)_ = 0.033) higher, respectively, than those from ‘high educational backgrounds’ (Table 3 and Figs. 4–6).

**Table 3:** BMI tracking between ages 11–42 years in women by SEP group (defined using parental education) and cohort, estimated using quantile regression

Cohort											
	1946 NSHD (*n* = 1221)				1958 NCDS (*n* = 3887)				1970 BCS (*n* = 2729)		
	*β*	95% CI		*p*_(diff to ‘High’)_	*β*	95% CI	*p*_(diff to ‘High’)_		*β*	95% CI	*p*_(diff to ‘High’)_
Quantile 0.1											
High education (ref)	0.36		0.23; 0.50	–	0.22	0.17; 0.28		–	0.16	0.09; 0.23	–
Middle education	0.26		0.16; 0.35	0.421	0.22	0.17; 0.28		0.965	0.19	0.13; 0.25	0.39
Low education	0.23		0.13; 0.34	0.105	0.23	0.18; 0.27		0.988	0.22	0.18; 0.27	0.134
Quantile 0.3											
High education (ref)	0.36		0.26; 0.47	–	0.27	0.20; 0.34		–	0.26	0.20; 0.33	–
Middle education	0.37		0.26; 0.48	0.779	0.26	0.19; 0.33		0.976	0.22	0.17; 0.28	0.297
Low education	0.32		0.25; 0.40	0.494	0.31	0.25; 0.36		0.43	0.29	0.22; 0.36	0.628
Quantile 0.5											
High education (ref)	0.41		0.28; 0.54	–	0.28	0.18; 0.39		–	0.33	0.25; 0.41	–
Middle education	0.53		0.38; 0.67	0.305	0.36	0.28; 0.45		0.209	0.31	0.24; 0.38	0.654
Low education	0.45		0.33; 0.56	0.614	0.44	0.40; 0.48		0.002	0.39	0.33; 0.46	0.269
Quantile 0.7											
High education (ref)	0.47		0.30; 0.64	–	0.49	0.34; 0.64		–	0.36	0.25; 0.46	–
Middle education	0.75		0.50; 0.99	0.032	0.53	0.42; 0.63		0.71	0.45	0.35; 0.54	0.185
Low education	0.6		0.45; 0.76	0.181	0.55	0.50; 0.59		0.413	0.54	0.48; 0.62	0.003
Quantile 0.9											
High education (ref)	0.64		0.33; 0.95	–	0.62	0.47; 0.77		–	0.48	0.31; 0.65	–
Middle education	1.00		0.72; 1.41	0.067	0.65	0.53; 0.77		0.997	0.53	0.36; 0.70	0.73
Low education	0.76		0.53; 0.98	0.605	0.74	0.66; 0.81		0.522	0.7	0.62; 0.77	0.033

Analyses substituting parental education with father’s social class revealed a similar pattern to that observed using educational background. There was no pattern of SEP inequalities in BMI tracking in the 1946 and 1958 cohorts (i.e. overlapping lines for ‘low’ and ‘high’ social class groups). In the 1970 cohort however, a pattern of higher tracking in women from ‘low social class’ backgrounds was observed i.e. line for ‘low social class’ consistently higher than that for ‘high social class’), though the strength of evidence was weak (Supplementary Table 3 and Supplementary Figs. 4–6).

## Discussion

In this exploratory analysis in three British birth cohorts, with data from over 15,500 participants spanning from ages 11–42 years (1957–2012), we observed higher child-adulthood tracking at the higher quantiles of the BMI distribution. While the strength of this tracking did not appear to systematically differ by socioeconomic group, there was suggestive evidence that tracking was stronger in lower socioeconomic groups among the 1970 cohort, especially in women.

The estimates of BMI tracking between 11–42 years at the median quantile in each of the three cohorts used in this study were similar to that reported by Bayer et al. [12] for a similar follow-up period, in their meta-analysis of BMI tracking over the life course (*r* = 0.4) [12]. Furthermore, the meta-analysis also supports our finding that the strength of tracking varies across the BMI distribution. Specifically, the meta-analysis observed that for each unit increase in average study BMI, tracking correlation coefficients increased by 0.018. The high prevalence of obesity in today’s children, alongside the finding that tracking is stronger at the upper end of the BMI distribution, suggests that a high proportion of children are likely to track in this weight classification throughout life and thus are likely to experience increased exposure to a range of cardiovascular risk factors and ultimately, an increased burden of non-communicable diseases [30–32].

A limited number of studies have looked at SEP inequalities in BMI tracking, all of which have been limited to a short period of the life course [23–25]. Our study however, based on a large sample from three nationally representative British cohorts, contributes novel evidence by revealing that the SEP association may persist over a much greater period of the life course. Kristiansen et al. [25] examined BMI tracking from birth to 7 years in 3771 children in the Norwegian Mother and Child Cohort Study (MoBa) and observed that children with parents with higher levels of education had lower odds being in the highest tertile of ponderal index at birth and overweight/obese at 7 years [25]. Kristensen et al. investigated the tracking of cardiovascular risk factors in 384 Danish school children aged between 8–16 years. In line with our findings, a higher BMI tracking coefficient was observed in the lower SEP group (defined according to mother’s occupational class) compared to high, with estimated tracking coefficients of 0.75 and 0.70, respectively. However, as the difference did not achieve statistical significance at *p* < 0.05, the authors concluded that SEP does not affect tracking [24]. Furthermore, a larger study (*n* = 4243) of overweight/obesity in a sample of children followed up between seven and 15 years of age, observed that children from lower SEP categories were almost half as likely to have a normal BMI at age 15 if previously classified as overweight/obese at age seven, compared with children from higher SEP groups [33]. Finally, a study in Austrian adults (mean age 42 years) over a period of 15 years also observed an increased odds of being classified as obese at successive measurements in ‘blue collar’ (OR = 1.11; 95% CI: 0.99–1.24, *p* = 0.07) and ‘self-employed’ (OR = 1.21; 95% CI: 0.97–1.50, *p* = 0.09) workers, relative to those employed in ‘white collar’ professions [23]. Taken together, these studies support our finding of stronger tracking in those from lower SEP groups. Unfortunately, none of the above studies investigated whether SEP inequalities in tracking differed according to sex and are thus unable to support our finding of a more consistent association between SEP and BMI tracking in women. While it has been shown that tracking of weight status is stronger in women [34–36], alongside stronger inverse associations between SEP and BMI in women [19, 21], further studies are required to replicate our finding of greater SEP inequalities in BMI tracking in women.

Socioeconomic inequalities in BMI have been demonstrated previously in the cohorts used in this study [6, 19, 21]. Despite a more recent study observing that SEP disparities were not apparent in childhood BMI (age 11 years) in these cohorts [22], it was observed that childhood BMI SEP inequalities were manifest in a more recently born cohort of children (Millennium Cohort Study (MCS)). Furthermore, SEP inequalities widened from childhood-to-adolescence (age 15 years) and were greater at the higher end of the BMI distribution. The finding that SEP inequalities in BMI (i.e. low SEP children more likely to be at higher end of distribution) are apparent earlier in the life course in more recent cohorts, alongside our findings that tracking may be stronger in lower SEP groups and at the higher end of the BMI distribution, suggests SEP inequalities in BMI tracking may be even larger in more contemporaneous cohorts, as children from low SEP groups are disproportionately located at the higher end of the BMI distribution at the outset. As has been argued previously [33], children from low SEP groups may therefore face a double burden of overweight/obesity, with an increased likelihood of becoming overweight/obese in childhood [18], alongside a reduced likelihood of regaining a normal BMI in later life.

SEP inequalities in the adoption of poorer lifestyle behaviours (e.g. higher fast food intake [37, 38], lower consumption of fruit and vegetables [39], breakfast skipping [40], excess screen time [41] and reduced physical activity [42]) are apparent in childhood and adolescence, with those from lower SEP groups displaying worse profiles. Differences in these health-related behaviours are thought to be driven by educational and economic inequalities in the ability to engage in health-promoting behaviours [43]. For example, the lower cost of energy dense, nutrition poor foods compared to fresh fruit and vegetables alongside the higher prevalence of fast food restaurants [44] and lower prevalence of recreational resources [45] in low SEP neighbourhoods are likely to contribute to the SEP disparities in these behaviours. A low SEP therefore reduces the ability of these individuals to engage in the positive health-related behaviours which may ameliorate an adverse childhood BMI, instead contributing to a stronger tracking of the adverse weight-related behaviours in low SEP groups [46].

There have been calls for interventions which target the environment in which these adverse health-related behaviours occur [47]. Accordingly, the UK government has recently introduced several population level policy interventions aimed at tackling the high rates of childhood overweight and obesity, outlined in their ‘Childhood Obesity: a plan for action’ 2016 guideline [48]. These include a call to reduce the sugar content within food by 20% by 2020 [49] and the introduction of a Soft Drinks Industry Levy (‘Sugar Tax’) [50], with the aim of getting producers to reduce the sugar contents of drinks. An obvious consequence of these tax-based approaches is a likely increase in price passed onto the consumer, which may have differential effects across SEP groups. Socioeconomic disparities in the effectiveness of public health interventions are well known, with a number of reports suggesting that interventions may actually increase disparities in health between these groups [51–55]. This has been observed in particular for individualistic interventions which place the responsibility on the individual [56, 57] and includes interventions aimed at promoting healthy eating [58–60]. More socioeconomically equitable results have, however, been observed for interventions acting at the more legislative and regulatory levels. For example, a systematic review of looking at the socioeconomic inequalities in the impact of interventions for the promotion of healthy eating observed that interventions based on taxation and subsidisation of foods were the most likely to reduce inequalities as these interventions preferentially improved healthy eating outcomes in people of lower SEP [61]. Nonetheless, as the prevalence of obesity is continuing to rise in many HICs, further interventions are required.

### Strengths

A key strength of this paper is the use of harmonised socioeconomic and BMI data across the three national birth cohorts, resulting in an analysis which was based on a much larger sample size than previous studies (e.g. the average sample size in the studies meta-analysed by Bayer et al. was ~1150 [12]). Furthermore, the fact that these cohorts are nationally representative cohorts suggests are findings are likely to be generalisable to the underlying population. Finally, by performing cohort-stratified analyses, we were able to compare cohort differences in tracking and its modification by sex and SEP. We repeated the analyses with another indicator of SEP, meaning we were able to establish the consistency of findings based on both education and social class indicators. A further strength of the paper is the assessment of BMI tracking using quantile regression, with the findings suggesting that while moderate tracking from adolescence-adulthood exists, it is largely driven by those at the higher end of the adult BMI distribution.

### Limitations

The use of BMI means we are unable to speculate as to the extent to which SEP disparities in tracking reflect inequalities in fat or fat-free mass tracking, which are likely to be associated with different longer-term risk profiles. The use of a complete case analysis may have introduced a selection bias into the observed estimates. All of the included studies experienced attrition which has been shown to be more extensive in those from lower SEP groups and/or with higher BMI [62, 63], meaning we may have inadvertently selected a more socioeconomically advantaged and thinner sample which as a result, may have led to us underestimate the observed SEP association.

## Conclusion

Childhood–adulthood BMI tracking appears to be stronger at higher quantiles of distribution, thereby demonstrating the difficulty that children with obesity have in normalising their weight status. We also found tentative evidence for a pattern of greater BMI tracking in lower (compared to higher) SEP groups in the more recent cohort, particularly in women and at the higher end of the BMI distribution. These findings need to be replicated in order to understand whether or not contemporaneous children with obesity from disadvantaged backgrounds, are more likely to be obese in adulthood than children who are equally obese but from advantaged backgrounds.

## Supplementary information

Supplementary table 1

Supplementary table 2

Supplementary table 3

Supplementary figure legends

Supplementary figure 1

Supplementary figure 2

Supplementary figure 3

Supplementary figure 4

Supplementary figure 5

Supplementary figure 6

## Data Availability

The statistical code for the analyses in this paper is available upon request from the corresponding author.
