# Supplementary material for: Socioeconomic inequalities in childhood-to-adulthood BMI tracking in three British birth cohorts
Source: Int J Obes (Lond). 2019 Jun 5;44(2):388–98. doi: 10.1038/s41366-019-0387-z (PMC6997121; doi:10.1038/s41366-019-0387-z)
Supplement: Supplementary file 4 — Supplementary figure legends [file 41366_2019_387_MOESM4_ESM.docx]

**Supplementary figure legends**

Supplementary figure 1: BMI tracking in high and low SEP groups (defined using father’s occupational social class) between ages 11-42 years in males in the 1946 cohort (n=1163)

Supplementary figure 2: BMI tracking in high and low SEP groups (defined using father’s occupational social class) between ages 11-42 years in males in the 1958 cohort (n=3734)

Supplementary figure 3. BMI tracking in high and low SEP groups (defined using father’s occupational social class) between ages 11-42 years in males in the 1970 cohort (n=2536)

Supplementary figure 4. BMI tracking in high and low SEP groups (defined using father’s occupational social class) between ages 11-42 years in females in the 1946 cohort (n=1148)

Supplementary figure 5. BMI tracking in high and low SEP groups (defined using father’s occupational social class) between ages 11-42 years in females in the 1958 cohort (n=3740)

Supplementary figure 6. BMI tracking in high and low SEP groups (defined using father’s occupational social class) between ages 11-42 years in females in the 1970 cohort (n=2677)
