# Supplementary figures and images for: Socioeconomic inequalities in childhood-to-adulthood BMI tracking in three British birth cohorts
Source: Int J Obes (Lond). 2019 Jun 5;44(2):388–98. doi: 10.1038/s41366-019-0387-z (PMC6997121; doi:10.1038/s41366-019-0387-z)

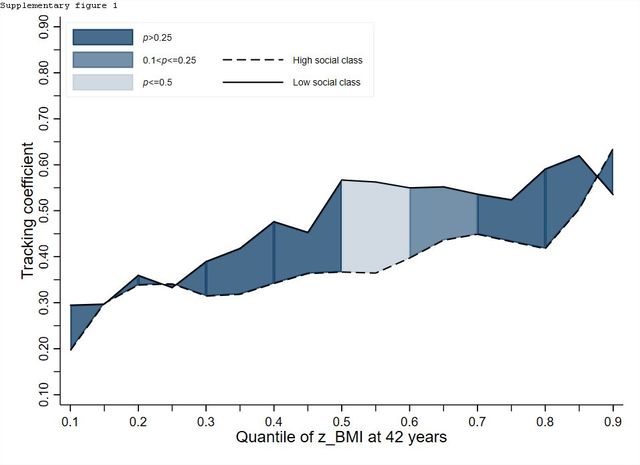

Supplement: Supplementary file 5 — Supplementary figure 1 [file 41366_2019_387_MOESM5_ESM.jpg]

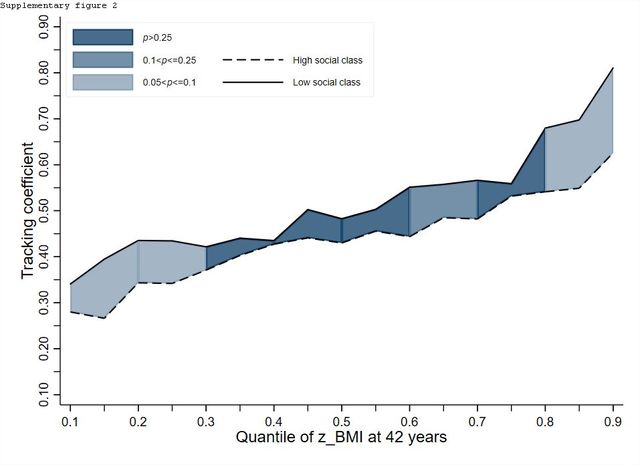

Supplement: Supplementary file 6 — Supplementary figure 2 [file 41366_2019_387_MOESM6_ESM.jpg]

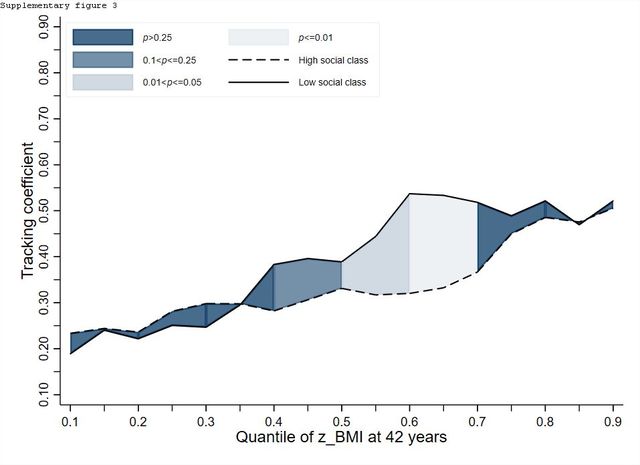

Supplement: Supplementary file 7 — Supplementary figure 3 [file 41366_2019_387_MOESM7_ESM.jpg]

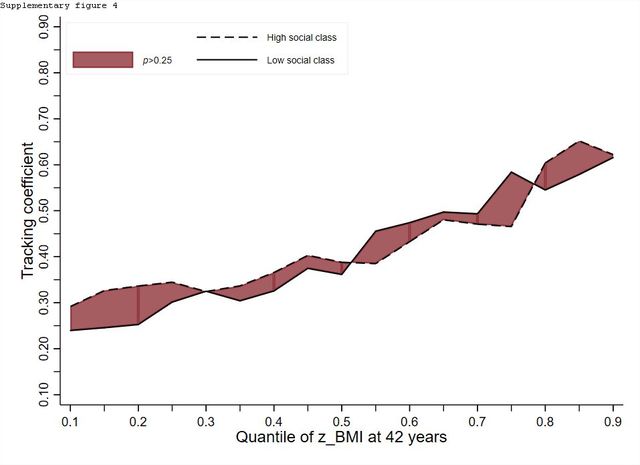

Supplement: Supplementary file 8 — Supplementary figure 4 [file 41366_2019_387_MOESM8_ESM.jpg]

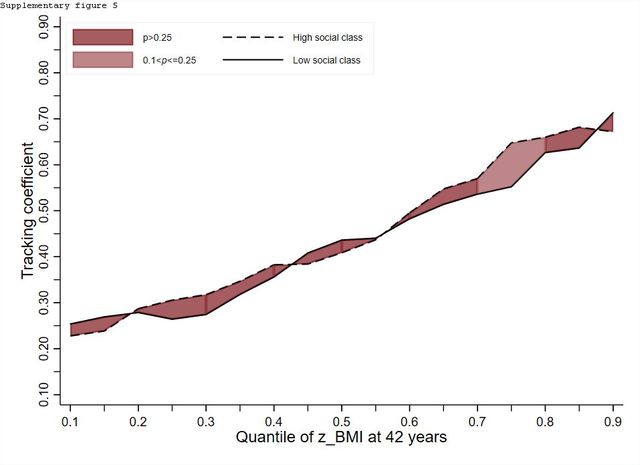

Supplement: Supplementary file 9 — Supplementary figure 5 [file 41366_2019_387_MOESM9_ESM.jpg]

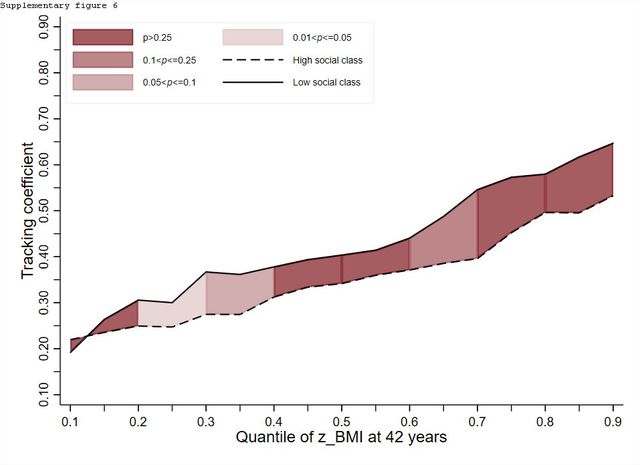

Supplement: Supplementary file 10 — Supplementary figure 6 [file 41366_2019_387_MOESM10_ESM.jpg]
